# Kin selection theory and the design of cooperative crops

**DOI:** 10.1111/eva.13418

**Published:** 2022-06-14

**Authors:** Jay M. Biernaskie

**Affiliations:** ^1^ Department of Crop Genetics John Innes Centre Norwich UK

**Keywords:** Darwinian agriculture, evolutionary agroecology, group selection, inclusive fitness, multilevel selection, tragedy of the commons

## Abstract

In agriculture and plant breeding, plant traits may be favoured because they benefit neighbouring plants and ultimately increase total crop yield. This idea of promoting cooperation among crop plants has existed almost as long as W.D. Hamilton's inclusive fitness (kin selection) theory, the leading framework for explaining cooperation in biology. However, kin selection thinking has not been adequately applied to the idea of cooperative crops. Here, I give an overview of modern kin selection theory and consider how it explains three key strategies for designing cooperative crops: (1) selection for a less‐competitive plant type (a ‘communal ideotype’); (2) group‐level selection for yield; and (3) exploiting naturally selected cooperation. The first two strategies, using artificial selection, have been successful in the past but suffer from limitations that could hinder future progress. Instead, I propose an alternative strategy and a new ‘colonial ideotype’ that exploits past natural selection for cooperation among the modules (e.g., branches or stems) of individual plants. More generally, I suggest that Hamiltonian agriculture—a kin selection view of agriculture and plant breeding—transforms our understanding of how to improve crops of the future.

## 1 INTRODUCTION

What is cooperation among crop plants? In social evolution theory, a cooperative trait has a beneficial effect on the reproductive success of others, and natural selection favours the trait because of this effect (Hamilton, 1964; West et al., 2007b). Yet, it is reasonable to extend this definition to include artificial selection in agriculture and plant breeding, where breeders may favour plant traits because they benefit neighbouring plants and ultimately increase total crop yield (e.g., fruit/grain production). In fact, one of the greatest successes in the history of agriculture—the introduction of shorter, less‐competitive cereal plants during the Green Revolution—is seen as a key example of artificial selection for cooperation (Denison et al., 2003; Denison, 2012; Weiner et al., 2010). In search of the next agricultural revolution, we could use a deeper understanding of how to get more cooperation into crops.

The idea of promoting cooperation among crop plants has actually existed almost as long as social evolution theory. In the 1960s, C.M. Donald argued that in a genetically uniform crop, the ideal plant type (‘ideotype’) should be a weak competitor, such that plants will minimally interfere with their neighbours (Donald, 1968). Around the same time, W.D. Hamilton developed inclusive fitness theory (now widely known as kin selection theory) and clarified how cooperation can be favoured by natural selection (Hamilton, 1963, 1964, 1970). The theory showed formally that a trait like reduced competitiveness can evolve more easily when interacting individuals share genes for the trait (i.e., there is high genetic relatedness in the population). Kin selection theory is now widely used to explain cooperation in biology, and the generality of the theory—including its equivalence to other theoretical approaches in social evolution—has been firmly established (Bourke, 2011; Frank, 1998, 2013; Gardner et al., 2011; Grafen, 1984; Hamilton, 1975; Marshall, 2011, 2015; Queller, 1992a,b; Rousset, 2004; Wade, 1985; West et al., 2007a).

However, our modern understanding of kin selection has not been adequately applied to the idea of cooperative crops. For example, Donald (1981) described the design of a less‐competitive or “communal” ideotype as “almost diametrically opposed to natural selection”, without recognizing any connection to kin selection. It has been more common to explain cooperation among crop plants as a result of group‐level selection, which is only rarely described in kin selection terms (Montazeaud et al., 2020). More often, group selection is described as an unnatural process and/or distinct from kin selection (Dension et al., 2003; Harper, 1981; Murphy et al., 2017; Weiner, 2019; Weiner et al., 2010, 2017). In other cases where kin selection thinking is applied to crop plants, the focus has been on kin discrimination/recognition—the possibility that plants recognize and cooperate with genetically similar neighbours but compete with dissimilar ones (Anten & Chen, 2021; Bais, 2018; Freville et al., 2019; Murphy et al., 2017). Yet, kin recognition is not a requirement for kin selection, and most cooperative traits in crop plants—like reduced height caused by mutant dwarfing alleles (Peng et al., 1999)—may be indiscriminate (expressed irrespective of neighbour identity). Focussing on discriminate traits hides the more fundamental role of kin selection theory for explaining all types of cooperation among crop plants.

Here, I use kin selection theory to help explain the historical successes and future potential in designing cooperative crops. My aim is to challenge the view of cooperative crop plants as ‘unfit’ or ‘impaired’ (Donald, 1981; Weiner, 2019); instead, a kin selection perspective sees the ideal cooperative crop plant as finely adapted to an environment with high genetic relatedness. To explain this perspective, I first give an overview of kin selection theory as a general statement of natural selection, and I illustrate its connection to multilevel (group) selection. I then examine how the theory can help to explain and refine strategies in plant breeding, including the design of communal ideotypes and group selection for yield. Finally, I argue that rather than trying to emulate kin selection, we could instead exploit cases where natural selection has already favoured cooperative plants. I suggest an alternative strategy and a new ideotype that could harness the full power of kin selection for cooperative crop plants.

## 2 THEORETICAL OVERVIEW

In this section, I present an overview of the most general version of kin selection theory, following Queller (1992a) and Gardner et al. (2011). The purpose of the theory is not to make specific predictions but to draw connections between different approaches in social evolution and, later, to the different ways of explaining cooperation among crop plants (for a defence of the general version of kin selection theory, see Birch & Okasha, 2015). To keep the models simple, I examine the effects of genes on individual reproductive success (fitness) but not the intermediate steps. For example, plant breeders may sometimes be interested in the effect of one plant's genes on the traits of its neighbours (indirect genetic effects, or IGEs; Bijma, 2014; Griffing, 1967; Moore et al., 1997). I do not consider IGEs explicitly in the models below; however, they are implicitly included because the models describe the total action of natural selection (Gardner et al., 2011).

The basic condition for natural selection to favour any trait is that the heritable component of the trait is positively correlated with individual fitness. This is captured by Price's equation for natural selection, which describes the change in the population average of a heritable trait *ΔE*(*g*) as the covariance of that trait *g* with relative fitness *w*:

(1) $$\mathrm{\Delta E}\left(g\right)=\mathrm{cov}\left(w,g\right)=\beta \left(w,g\right)\;\mathrm{var}\left(g\right)$$

(Gardner, 2020; Price, 1970). In this Equation, *var*(*g*) is the heritable variance in the trait and *β*(*w,g*) is the least squares linear regression coefficient for the relationship between individual fitness and the heritable trait (the selection gradient). Hence, as long as there is genetic variation in the population (*var*(*g*) > 0), larger values of the focal trait will be favoured when the selection gradient is positive (*β*(*w,g*) > 0).

**FIGURE 1:**
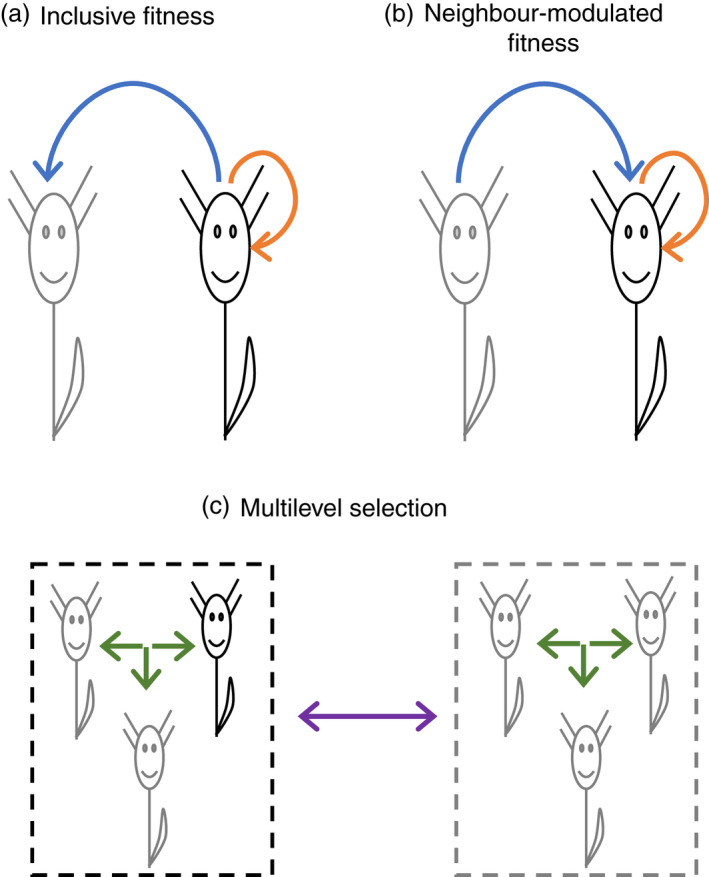
Different ways of partitioning natural selection in a plant population. (a) The inclusive fitness approach considers the direct effect from a focal plant's own heritable trait on its own fitness (orange arrow) and an indirect effect on the fitness of its neighbours (blue arrow). (b) The neighbour‐modulated fitness approach considers the direct fitness effect from a focal plant's own heritable trait (orange arrow) and an indirect effect from that same trait in its neighbours (blue arrow). (c) The multilevel selection approach considers within‐group effects (green arrows) and among‐group effects (purple arrows). Adapted from West & Gardner (2013)

I consider three different ways of partitioning natural selection acting on a heritable plant trait with social effects (Figure 1). The heritable trait could be height or leaf size, for example, with larger values having a harmful effect on neighbours (owing to increased shading) and smaller values having a beneficial effect on neighbours. The different ways of partitioning selection correspond to different, but all valid, approaches in social evolution theory—they are simply different ways of looking at the same process (Marshall, 2011; Wenseleers et al., 2010; West & Gardner, 2013). The first two approaches are different perspectives of kin selection theory, and the third approach is multilevel selection theory, which can also be understood in the language of kin selection.

### 2.1 Kin selection

The first and perhaps most familiar perspective of kin selection is the ‘inclusive fitness’ approach (Hamilton, 1964). This approach partitions selection into effects that come from the focal plant's heritable trait only (Figure 1a). This is because there are two ways for a heritable trait to be positively correlated with an individual's inclusive fitness: it may directly benefit the focal plant (direct fitness effect), and it may benefit the focal plant's neighbours who share genes for the trait (indirect fitness effect). In the terms of least squares multiple regression, this can be expressed as:

(2) $$\begin{array}{c} \beta \left(w,g\right)=\underset{\text{direct}\;}{\underbrace{\beta \left(w,g\;|\;g^{\prime }\right)}}+\underset{\text{indirect}}{\underbrace{\beta \left(w^{\prime }\;,g|g^{\prime }\right)\;\beta \left(g^{\prime }\;,g\right)}}, \end{array}$$

where *β*(*w,g*|*g'*) = *−c* is the effect of a focal plant's own heritable trait on its own fitness, holding *g'* fixed; *β*(*w',g*|*g'*) = *b* is the effect of a focal plant's heritable trait on the fitness of its neighbours; and *β*(*g',g*) = *r* is the statistical association between the heritable trait in a focal plant and its neighbours (the kin selection coefficient of genetic relatedness). The trait will therefore be favoured when –*c* + *br*   > 0, which is Hamilton's rule of kin selection (Hamilton, 1963, 1964).

The second perspective of kin selection is the ‘personal’ or ‘neighbour‐modulated’ fitness approach (Frank, 1998; Hamilton, 1964; Rousset, 2004; Taylor & Frank, 1996). This approach partitions selection into effects from a focal plant's own heritable trait and from that same trait expressed in neighbouring plants (Figure 1b). This is because there are two ways for a heritable trait to be positively correlated with personal fitness: it may directly benefit the focal plant (direct fitness effect, as above), and it may be expressed by the focal plant's neighbours, with average heritable trait *g'*, in a way that benefits the focal plant (indirect fitness effect). This can be expressed as:

(3) $$\begin{array}{c} \beta \left(w,g\right)=\underset{\text{direct}\;}{\underbrace{\beta \left(w,g\;|\;g^{\prime }\right)}}+\underset{\text{indirect}}{\underbrace{\beta \left(w,g^{\prime }|\;g\right)\;\beta \left(g^{\prime },g\right)}}\;, \end{array}$$

where the new term is *β*(*w,g'*|*g*) = *b*, the effect of the mean heritable trait of a focal plant's neighbours on the focal plant's fitness, holding *g* fixed. Hence, like the inclusive fitness approach, the neighbour‐modulated fitness approach partitions selection into direct and indirect effects, and it shows that a heritable trait will be favoured when Hamilton's rule is satisfied (−*c* + *br* >0).

The key difference between the neighbour‐modulated and inclusive fitness approaches is the directionality of the indirect fitness effect (*br*) and the corresponding interpretation of relatedness (Frank, 1997, 1998; West & Gardner, 2013). Consider, for example, a heritable trait that benefits neighbours (*b* > 0). The inclusive fitness perspective treats the benefit as going from the focal plant to its neighbours, and relatedness can be understood as the extent to which the focal plant values the reproduction of its neighbours because they share genes. In contrast, the neighbour‐modulated fitness perspective treats the benefit as coming to the focal plant from its neighbours, and relatedness can be understood as the extent to which the focal plant and its neighbours will have similar heritable traits. Higher relatedness means that a cooperative focal plant can transmit more genes for cooperation through the reproduction of its neighbours (inclusive fitness view) or that a focal plant will have neighbours that more closely mirror its tendency for cooperation (neighbour‐modulated fitness view).

### 2.2 Multilevel selection in kin selection terms

A slightly modified neighbour‐modulated fitness partitioning is helpful for comparison to the multilevel selection approach. Specifically, we can modify Equation 3 above to consider the focal plant's fitness being affected by the average heritable trait *G* of the whole group, including the focal plant and its neighbours, rather than the average trait *g'* of the focal plant's neighbours only. In this case, the selection gradient can be expressed as:

(4) $$\beta \left(w,g\right)=\beta \left(w,g|G\right)+\beta \left(w,G|g\right)\;\beta \left(G,g\right)\;,$$

where *β*(*w,g*|*G*) is the effect of the focal plant's own heritable trait on its own fitness, holding *G* fixed; *β*(*w,G*|*g*) is the effect of the average heritable trait of the whole group on the focal plant's fitness, holding *g* fixed; and *β*(*G,g*) = *R* is ‘whole‐group’ relatedness, a measure of the average genetic similarity between the focal individual and all individuals in the group, including itself (hence, *R* is always positive because the focal plant's similarity to itself is 1). A consequence of this modification is that the fitness effects in Equation 4 no longer match the –*c* and *b* terms of Hamilton's rule. Nevertheless, this ‘whole‐group’ formulation is a useful model for many social traits (Pepper, 2000), and it allows for a simpler relationship with multilevel selection models.

The multilevel selection (or ‘group selection’) approach partitions the total action of natural selection into among‐ and within‐group effects (Gardner, 2015; Hamilton, 1975; Price, 1972) (Figure 1c). The among‐group effect describes the relationship between the heritable trait and fitness at the group level (i.e., the collective fitness of individuals in the group), whereas the within‐group effect describes the relationship between the heritable trait and fitness within groups. The relative strength of these effects depends on how much heritable variation exists among groups relative to within groups. Following Lehtonen (2016), this can be expressed in kin selection terms as:

(5) $$\begin{array}{c} \beta \left(w,g\right)=\underset{\text{among‐group}}{\underbrace{(\beta \left(w,g\;|\;G\right)+\beta \left(w,G\;|\;g)\right)\;R\;}}+\underset{\text{within‐group}}{\underbrace{\beta \left(w,g\;|\;G\right)\;\left(1-R\right)}}, \end{array}$$

which is simply a rearrangement of Equation 4 (illustrating an equivalence between kin and multilevel selection approaches). In this equation, the whole‐group coefficient of relatedness *R* determines the relative strength of among‐group versus within‐group effects. Higher whole‐group relatedness means that the plants in a group have more similar heritable traits (hence, more genetic variation among groups relative to within groups), and this promotes cooperative traits that increase group‐level fitness.

### 2.3 Summary and application to cooperative crops

All three of the theoretical approaches above illustrate how higher genetic relatedness promotes cooperation among plants (Table 1). The general theory also clarifies that relatedness is simply a measure of statistical association between the heritable traits of neighbouring plants—that is, when a focal plant has a heritable trait that is higher/lower than the population average, relatedness measures the extent to which the heritable traits of its neighbours will be higher/lower than the population average. Yet, the different theoretical approaches provide different ways of interpreting relatedness and explaining selection for cooperation. Depending on the context, one approach may provide a more intuitive explanation than another (Birch & Okasha, 2015; Frank, 1997).

**TABLE 1:** The kin selection coefficient of relatedness is central to the evolution of cooperation

Theoretical approach	Cooperation promoted by high relatedness?	Interpretation of high relatedness
Inclusive fitness	Yes	Genes can be passed on through neighbours
Neighbour‐modulated fitness	Yes	Neighbours have similar heritable traits
Multilevel selection	Yes	High genetic variation between groups

So how does the general theory of kin selection help to understand strategies for designing cooperative crops? Crucially, it suggests that all strategies will be connected by the fundamental principle that cooperation is promoted by high relatedness. It also tells us that crop plants do not need special mechanisms to detect the genetic similarity of neighbours—for example, plant breeders create high relatedness by simply grouping together plants with the same genotype or a similar genetic tendency for a focal trait. Moreover, the general theory offers different ways of explaining selection for cooperative crop plants. To explain cooperative adaptations evolved by natural selection, it can be helpful to think of individuals as ‘striving’ to maximize their inclusive fitness (West & Gardner, 2013). In contrast, the goal of plant breeders is more aligned with maximizing neighbour‐modulated fitness or group‐level fitness, which leads to different explanations.

In the following sections, I examine how kin selection theory can help to explain three strategies for designing cooperative crops: communal ideotype breeding, group selection for yield, and exploiting naturally selected cooperation. The first two strategies use artificial selection methods that have not usually been linked to kin selection. I discuss areas where kin selection theory could help to refine these methods, and I also consider their limitations. To address the limitations, I propose an alternative strategy based on exploiting past natural selection for cooperative plants.

## 3 ARTIFICIAL SELECTION FOR COOPERATIVE CROP PLANTS

The predominant method for increasing cooperation among crop plants has been through artificial selection in plant breeding. This includes major breeding successes during the Green Revolution and more recent advances using components of communal ideotype breeding (selecting for traits of individual plants) and group selection for yield (selecting whole groups of plants). The explicit aim of plant breeders has probably not been to increase cooperation or to emulate kin selection. Yet, I argue here that kin selection theory explains why these breeding strategies have been so successful. Hence, the key examples of past selection for cooperative crop plants can be seen as examples of applied kin selection theory. An explicit recognition of how kin selection theory is related to plant breeding could help to intentionally select for more cooperative crop plants.

Both communal ideotype breeding and group selection for yield address the problem known as a ‘tragedy of the commons’ in evolution (Box 1; Anten & Vermeulen, 2016; Rankin et al., 2007). The problem occurs when traits that give an individual a competitive advantage also have a negative effect on its neighbours (e.g., a taller plant getting more light and shading its neighbours), so higher average competitiveness leads to lower group productivity. Artificial selection for cooperative crop plants is often thought of as ‘reversing’ natural selection for individual competitiveness (Anten & Vermeulen, 2016; Denison, 2012; Donald, 1981). In contrast, kin selection theory tells us how natural selection itself can resolve the tragedy of the commons (Box 1), which could in turn help to understand and refine artificial selection for cooperative crop plants.

Box 1 **Kin selection and the tragedy of the commons**

A tragedy of the commons arises because of the tension between individual self‐interest and group productivity. Following Frank (1994, 1998), a simple model of this tension supposes that an individual's fecundity (e.g., seed production) is proportional to *f = x*/*y* (1–*y*), where I consider *x* to be a focal plant's competitiveness (a heritable trait ranging from 0 to 1) and *y* to be the average competitiveness of the focal plant and its interacting neighbours. In this function, the first component *x*/*y* represents the focal plant's relative competitiveness for contested resources, and the second component (1–*y*) represents the average productivity of the whole group, which declines with higher average competitiveness. I assume implicitly that plant density is high and that group size (number of interacting neighbours) remains constant.

This model includes no potential for competition due to local seed dispersal. It is well known that local dispersal can inhibit cooperation if helping related neighbours to produce more offspring also increases competition among offspring for space in the next generation (Taylor, 1992). In contrast, I assume here that an individual's neighbour‐modulated fitness is simply equivalent to its fecundity (*w*[*x,y*] = *f*), which is a reasonable assumption for plants with effective seed dispersal. The model is also relevant to natural selection occurring in crops or breeding populations, in cases where seeds are harvested from the plants and then ‘dispersed’ to establish the next generation.

I use the method from Taylor and Frank (1996) to analyse natural selection acting on individual competitiveness. This method derives the selection gradient *β*(*w,x*) by assuming that the focal plant's competitiveness *x* varies only slightly from the population average competitiveness *z*. It is then possible to approximate fitness effects in the selection gradient with partial derivatives: *β*(*w,x*|y*)* ≈ ∂*w*/∂*x*|_*x = y = z*_ and *β*(*w,y*|*x*) ≈ ∂*w*/∂*y*|_*x = y = z*_. Following Equation 4 and Equation 5 in main text, the selection gradient can be written as

(6) $$\beta \left(w,x\right)\approx \left(1-z\right)/z+\left(-1/z\right)\;R$$

or

(7) $$\beta \left(w,x\right)\approx \underset{\text{among‐group}}{\underbrace{\left(-1\right)\;R\;}}+\underset{\text{within‐group}}{\underbrace{\left(\left(1-z\right)/z\right)\;\left(1-R\right)}}.$$

In Equation 6, the first term on the right‐hand side is the focal plant's benefit of being relatively competitive, and the second term is its productivity cost of higher competitiveness, which gets larger as whole‐group relatedness *R* increases (as neighbours more closely mirror the focal plant's own competitiveness). Similarly, Equation 7 shows that increasing whole‐group relatedness gives less weight to within‐group selection for competitiveness and more weight to among‐group selection, which always inhibits individual competitiveness. Accordingly, the optimal individual competitiveness declines as relatedness increases. Specifically, by finding the point at which directional selection stops (*β*(*w,x*) = 0), both approaches above predict a candidate evolutionarily stable (optimum) competitiveness of z* = 1 – *R*. In the extreme, when *R* = 1, natural selection favours the group optimum trait value z* = 0, where group productivity is maximized and the tragedy of the commons is completely resolved.

### 3.1 Communal ideotype breeding

**FIGURE 2:**
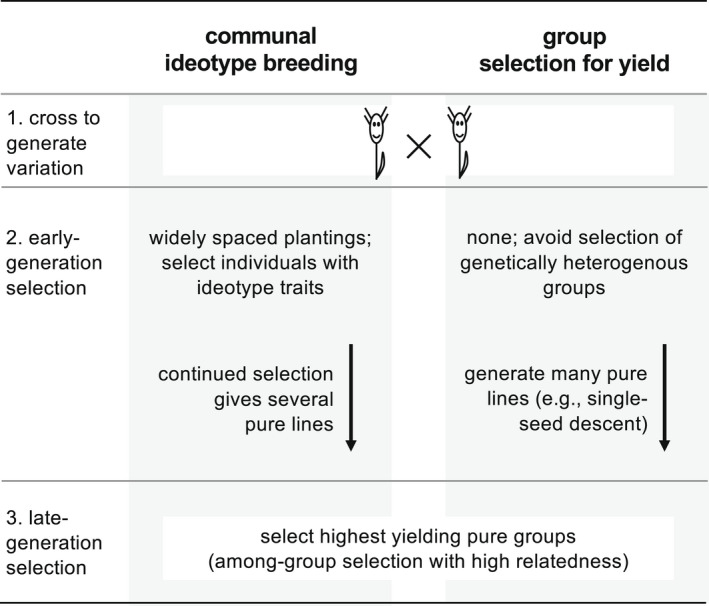
Two types of artificial selection for cooperative crop plants. This schematic reflects a general best practice for self‐pollinated crops, where homozygous pure lines are generated by repeated selfing following an initial cross

The aim of communal ideotype breeding is to design the traits of individual plants to be maximally productive in a high‐density crop environment (Donald, 1968, 1981). Specifically, Donald (1968) predicted that weak individual competitiveness (e.g., few stems/plant; short height; and small, erect leaves) will be optimal in dense groups where neighbours are also weak competitors (genetically uniform, or ‘pure’ groups). Selection for a communal ideotype is compatible with a pedigree breeding approach (Bos & Caligari, 2008), in which: (i) following a cross, plants are grown with wide spacing and breeders may select individual plants with ideotype traits conferring reduced competitiveness; and (ii) the selected genotypes are later grown in high‐density pure groups and selected based on group yield (Figure 2). A key example of communal ideotype breeding is the design of dwarfed, Green Revolution rice varieties that are easily outcompeted by taller and more leafy types but yield more total grains when grown in pure groups (Jennings, 1964; Jennings & DeJesus, 1968).

The communal ideotype is consistent with predictions from kin selection theory about the optimal competitiveness of individual plants. In a tragedy of the commons scenario, natural selection can favour weaker competitors as relatedness increases (Box 1). This is because when relatedness is high, it is as if a plant has information that its neighbours' competitiveness will mirror its own (Frank, 1998) and, in this case, reduced competitiveness is the optimal (evolutionarily stable) solution. Similarly, if plant breeders know that plant types will be deployed in high‐density pure groups (high *R*), then the optimal type will be a weak competitor, as Donald (1968) predicted. Conversely, in crops made up of well‐mixed genotypes (low *R*), weak competitiveness is no longer evolutionarily stable, and so more‐competitive types can take over (Jennings & DeJesus, 1968; Knapp et al., 2020).

Kin selection theory could help to identify additional traits for a communal ideotype. The general theory is related to quantitative genetic methods that can be used to estimate the fitness effects of social traits in crops (‘social selection’ and ‘contextual’ analyses; Heisler & Damuth, 1987; Marin, 2021; McDonald et al., 2017; Wolf et al., 1999). Specifically, following Equation 3 or Equation 4, this can be done by relating the individual yield of focal plants to a heritable trait of focal plants and their neighbours and then estimating the appropriate partial regression coefficients. Following Equation 3, for example, a desirable communal ideotype trait will have a positive or only weakly negative direct fitness effect (*β*(*w,g*|*g'*)) and a large positive effect on the reproduction of neighbours (large *β*(*w,g'*|*g*)).

### 3.2 Group selection for yield

Group selection in plant breeding favours genotypes based on the collective yield of whole groups rather than the traits of individuals. This differs from communal ideotype breeding in that there is no direct selection for individuals with reduced competitiveness (Figure 2). However, individuals with less‐competitive traits are nevertheless expected to be favoured because these traits ultimately increase group productivity. Group selection is a major part of plant breeding because the total yield of pure groups is typically evaluated in the final stages of any breeding process (Murphy et al., 2017). A key example comes from post‐Green Revolution maize breeding, where group selection for yield has been the primary selection method and has apparently favoured several traits that reduce individual competitiveness, including smaller tassel size and more erect leaves (Duvick & Cassman, 1999).

The success of selection among genetically‐uniform groups is consistent with predictions from kin selection theory. In a tragedy of the commons scenario, among‐group selection for reduced competitiveness becomes more efficient as genetic relatedness increases (Box 1). This is because higher relatedness corresponds to greater genetic variation among groups relative to within groups. Conversely, as relatedness declines, within‐group selection for individual competitiveness increasingly works against group selection and lowers the collective productivity of groups. Similarly, in plant breeding, having more genetic variation within groups (lower *R*) means that group selection for yield is less able to favour group‐beneficial traits (Montazeaud et al., 2020).

Emphasizing the key role of genetic relatedness may help to improve the efficiency of group selection for yield. For example, Murphy et al. (2017) proposed that group selection should be applied earlier in the plant breeding process, rather than in the final stages only. However, in a segregating population following an initial cross, any groups that are created will likely contain significant genetic variation that could obscure group selection for yield. An alternative to early‐stage group selection is to first use single‐seed descent or doubled‐haploid methods to quickly generate many homozygous, pure lines (Bos & Caligari, 2008). This would ensure that subsequent group selection for yield always occurs in populations with maximal relatedness (Figure 2).

### 3.3 Limitations of artificial selection

Despite the potential for refining artificial selection for cooperative crop plants, there are several factors that could limit future progress. Firstly, there must be appropriate genetic variation for further reductions to the competitiveness of plants and sufficient time (in generations) to incorporate this variation into crops. For example, it has been a challenge to incorporate the desired traits into a communal ideotype (Donald, 1981; Marshall, 1991), and it has taken decades to make progress with group selection for yield (Duvick & Cassman, 1999). Another limitation is that the monoculture crops associated with less‐competitive plants can negatively impact biodiversity and ecosystem services, and they tend to be more susceptible to pests, disease and weeds than more diverse crops (Isbell et al., 2017; Reiss & Drinkwater, 2018; Tamburini et al., 2020). Overall, breeding for less‐competitive plants may not be the most timely or sustainable solution to the future demands of agriculture.

## 4 EXPLOITING NATURAL SELECTION FOR COOPERATIVE CROP PLANTS

An alternative and underappreciated strategy for designing cooperative crops is to exploit past natural selection. In contrast to the limitations of plant breeding, natural selection has had millions of years to test genetic variants and refine the adaptations of plants in nature. Therefore, if some plants have evolved in populations with high genetic relatedness (and have had mechanisms to avoid local competition among seeds; Box 1), then we can expect the potential for finely‐tuned adaptations for cooperation. In fact, naturally selected adaptations might even be more complex than the reduced individual competitiveness that can be achieved by plant breeding. From an inclusive fitness perspective, cooperative plants could sometimes sacrifice their own direct fitness to increase the fitness of closely related neighbours.

There are undoubtedly special cases where the ancestors of crop plants evolved in environments with high relatedness, suggesting where to look for cooperative adaptations. In the extreme, many plants grow by producing repeated, genetically identical modules that each have their own reproductive potential (e.g., fruit/grain‐bearing stems, branches or clonal ‘ramets’; Harper, 1981). Most cereal plants, for example, can grow to consist of many densely spaced branches (tillers) that each produce grains. In such cases where modules are physiologically integrated and clonally related (*r* = 1), natural selection could favour highly coordinated cooperation among modules to maximize the collective fitness of the individual (Gardner & Grafen, 2009; Sadras & Denison, 2009). For example, evidence suggests that the modules of individual plants cooperate by: dividing labour to acquire localized resources, sacrificing resources to share with others, and avoiding between‐module competition (Liu et al., 2016; Sadras & Denison, 2009). This suggests that one way to design a cooperative and productive crop is to create an environment that promotes large, highly modular plants with naturally‐evolved cooperation occurring among the modules within each plant.

**FIGURE 3:**
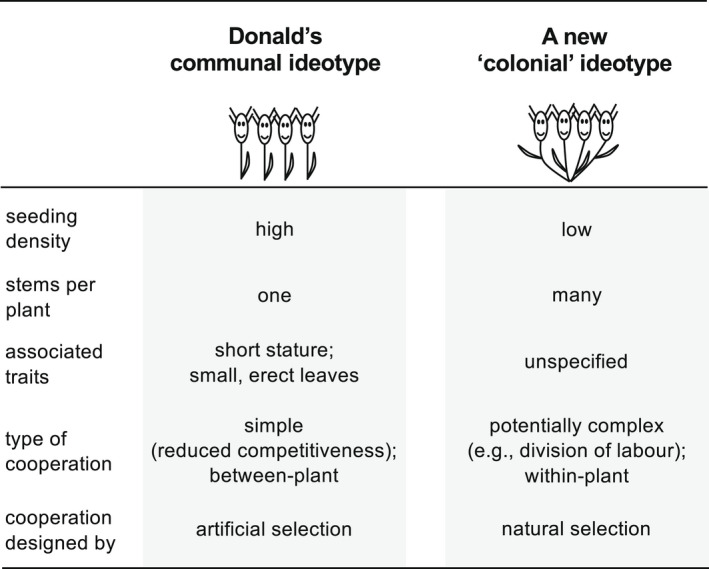
Alternative ideotypes for designing cooperative crops. The communal ideotype is a design for weakly‐competitive plants growing at very high density, ensuring complete exploitation of available resources. The colonial ideotype is for crops planted at a relatively low density, giving space for plants to expand by modular/clonal growth and express adaptations for cooperation among modules

To harness the potential for cooperation within highly modular plants, I propose a ‘colonial’ ideotype that stands in contrast to Donald's communal ideotype (Figure 3). As discussed above, the communal ideotype is designed for high‐density crops (many plants per unit area) and is meant to have only a single stem per plant (along with other traits associated with reduced individual competitiveness). This is because increased branching in a high‐density crop environment is expected to be harmful, owing to increased competition among neighbouring plants (Donald, 1968; Kokubun, 1988). In contrast, the colonial ideotype is for low‐density crops (few plants per unit area) and would be designed to have many stems or branches per plant. For example, breeders could select for genotypes that have a particularly high propensity for modular growth (e.g., high tillering capacity; Bilgrami et al., 2020; Haaning et al., 2020; Jiang et al., 2019). In a low‐density crop environment, increased branching is expected to be beneficial because: (i) plants will have space to expand by modular growth; and (ii) plants may have highly effective adaptations for cooperation among modules that will have evolved to maximize the collective fitness of the individual. Consequently, it is possible that crops made up of the colonial ideotype, with naturally‐selected cooperation within plants, could be more productive than crops made up the communal ideotype, with artificially‐selected cooperation between plants.

Further research is needed to determine whether a low‐density, colonial ideotype strategy could be superior to a high‐density, communal ideotype strategy. Relevant evidence from soybean crops suggests that a non‐branching type at high density can actually yield more total grains than a branching type at low density (Kokubun, 1988). On the other hand, there is emerging evidence that wheat crops sown at extremely low density, thereby encouraging extensive tillering, can achieve higher total grain yields than crops sown at a conventional density (Fischer et al., 2019; Fischer, 2020). Beyond yield considerations, using low‐density crops with minimal competition among plants could also make it easier to increase crop diversity—for example, by growing colonial ideotypes from different genotypes in the same crop (varietal mixtures; Wuest et al., 2021) or by growing different crop species among colonial ideotypes of a focal crop species (intercropping; Bourke et al., 2021). Moreover, it is possible that by sharing resources among modules, plants with a colonial ideotype will be more resilient to stresses and environmental variation. Overall, adopting the colonial ideotype could lead a radical shift towards crop designs that are more productive and sustainable than conventional monocultures.

## 5 CONCLUSION

This study contributes to a kin selection perspective of agriculture and plant breeding (Kiers & Dension, 2014), which could be called ‘Hamiltonian agriculture’. I suggest that kin selection thinking is crucial for understanding the different strategies involved in selecting for cooperative crop plants. The general theory helps to explain all of these strategies and connects them to the fundamental principle that cooperation is promoted by high genetic relatedness. Adopting a kin selection perspective transforms the view of plant breeding as ‘unnatural’ or necessarily working against natural selection; instead, breeding strategies can be refined with the aim of emulating kin selection for maximal cooperation. Given the limitations of artificial selection, however, perhaps the most important contribution of Hamiltonian agriculture will be to reconsider where naturally selected plant cooperation can be better exploited in crops of the future. Further research into the colonial ideotype could illustrate how kin selection thinking underlies a revolutionary way forward in sustainable crop design.

## CONFLICT OF INTEREST

I have no conflict of interest to declare.

## Data Availability

This manuscript contains no data.
